# Influence of Aldehyde-Based Modifiers on Rubber Asphalt: Properties, Deodorization Effect, and Mechanistic Analysis

**DOI:** 10.3390/polym18070799

**Published:** 2026-03-26

**Authors:** Honggang Zhang, Jiechao Lei, Hui Huang, Xiaowen Wang, Yongjun Meng, Pengkun Shao, Lihao Zeng

**Affiliations:** 1 Guangxi Key Laboratory of Road Structure and Materials, Guangxi Transportation Science and Technology Group Co., Ltd., Nanning 530007, China; cslgzhg@163.com (H.Z.); tina19840630@163.com (H.H.); wxw7778888@163.com (X.W.); 18377157451@163.com (P.S.); 13973437513@163.com (L.Z.); 2 College of Civil Engineering and Architecture, Guangxi University, Nanning 530004, China; hitmengyj@163.com; 3 Guangxi Transportation Science New Material Technology Co., Ltd., Nanning 530007, China

**Keywords:** crumb rubber modified asphalt, conventional properties, deodorization effect, deodorization mechanism, α-Amyl cinnamic aldehyde

## Abstract

A sustainable way to recycle used tires and improve the functionality of asphalt pavements is through the use of crumb rubber modified asphalt (CRMA). However, its application during high-temperature construction raises environmental and occupational health concerns due to the release of significant quantities of odorous and potentially harmful gases. Therefore, this study selected α-Amyl cinnamic aldehyde (ACA) as a deodorant and added it to CRMA at proportions of 0.5%, 1.0%, 1.5%, and 2.0% to prepare DCRMA. A number of common tests, such as softening point, ductility, penetration, Brookfield rotational viscosity, and segregation analysis, were used to evaluate the basic characteristics of the modified asphalt. A self-developed asphalt fume monitoring device was used to quantitatively analyze the changes in VOCs, H_2_S gas concentration, and solid particle content in the asphalt fumes to assess the deodorization effect of ACA on CRMA. Furthermore, the deodorization mechanism of ACA on CRMA was explored in depth using microscopic methods, such as fluorescence microscopy (FM) and Fourier transform infrared spectroscopy (FTIR). The findings demonstrated that ACA can increase the softening point and viscosity of CRMA while decreasing its penetration and ductility. The storage stability was optimal at a 1.0% ACA addition. Additionally, as the ACA content increased, the concentrations of VOCs, H_2_S gas, and solid particles in the asphalt fumes continued to decrease. FM results indicated that when the ACA content did not exceed 1.0%, it promoted the swelling degree of CR in the asphalt. FTIR results showed that ACA can reduce the characteristic peak intensity of CRMA. This study offers important technical references and practical support for the environmentally friendly use of CRMA.

## 1. Introduction

Every year, about 1.4 billion tires are used worldwide, and once their service life ends, they are discarded as waste, contributing significantly to environmental pollution [1]. To mitigate this issue, crumb rubber (CR), derived from waste tires, is commonly used as a modifier in asphalt to produce CRMA, which has been widely employed in road pavement construction [2]. In addition to lessening the negative effects of waste tire buildup on the environment, this method improves asphalt’s fatigue durability and tolerance to high and low temperatures, encouraging the sustainable use of waste tires [3]. However, during the high-temperature construction process, CRMA releases substantial quantities of odorous gases, which pose serious risks to the health of construction workers and negatively impact the environment [4]. Therefore, how to reduce the emission of harmful gases from CRMA during pavement construction and achieve green and sustainable development goals is a current research hotspot among scholars.

During the tire manufacturing process, the carbon–carbon double bonds in the CR molecular structure typically form stable vulcanized bonds through cross-linking reactions with vulcanizing agents under specific temperature conditions. This process promotes the generation of large amounts of sulfur compounds, which leads to a significant increase in the amount of sulfur compounds in rubber products. Therefore, more toxic and harmful gases are produced during the preparation, transportation, and paving of CRMA [5]. Among them, H_2_S, CO_2_, and C_3_H_8_ are the three major components with the highest concentrations [6]. It should be noted that while CO_2_ and C_3_H_8_ are not toxic, H_2_S is the main component responsible for the foul smell in CRMA mixtures. To reduce the emission of harmful gases from CRMA, researchers have made extensive explorations and attempts, among which the addition of deodorants is one of the common methods [7,8]. Zhao et al. [9] investigated the deodorizing effect of zinc stearate (ZS) on sulfurized gases emitted from CRMA and demonstrated that ZS effectively suppresses the desulfurization and degradation of CR. Similarly, Ortíz-Magán et al. [10] reported comparable findings, confirming the effectiveness of ZS in reducing sulfurized emissions. Furthermore, Niu et al. [11] evaluated the performance of three deodorants, ZS, molecular sieve (MS), and layered double hydroxides (LDHs), in mitigating sulfurized gas emissions from CRMA. Their study revealed that the combined application of these deodorants significantly inhibits the desulfurization and degradation of CR, reduces sulfur release, and effectively suppresses the emission of sulfurized gases. Additionally, Li et al. [12] found that the incorporation of plant alcohol promotes the aggregation of rubber powder, limiting its migration within the asphalt matrix. This aggregation process effectively reduces the desulfurization and degradation of CR, leading to a notable decrease in the amount of H_2_S emitted from CRMA fumes.

Nevertheless, the majority of recent research has ignored the volatile organic compounds (VOCs) in CRMA in favor of measuring H_2_S concentrations to evaluate deodorization effects. According to earlier research, VOCs include strong-smelling molecules, including dimethyl sulfide and methyl mercaptan, in addition to sulfur-containing compounds [12,13]. The two primary types of commonly utilized VOC inhibitors are volatile organic masking agents and inorganic porous adsorbents. To obtain effective adsorption, inorganic porous adsorbents usually need a large dose (>5 wt%), which can make them less compatible with matrix asphalt and lower asphalt performance [14]. Conversely, redox reactions or volatile masking are the main ways that volatile organic masking agents work [15]. However, these agents often lack long-term stability, and their reaction processes may generate new VOCs, ultimately failing to meet the desired performance requirements. Based on this, Meng et al. [16] studied the inhibitory effects of vanillin, citral, and α-Amyl cinnamic aldehyde on VOCs in SBS-modified asphalt and found that all three aldehyde modifiers can effectively reduce VOC concentrations and significantly enhance the high-temperature stability of asphalt, with α-Amyl cinnamic aldehyde showing the most significant effect.

Therefore, this study selected ACA as a deodorant to be added to CRMA to produce DCRMA, aiming to reduce the release of H_2_S and VOCs while maintaining the performance of asphalt. First, the conventional properties of DCRMA were systematically evaluated through penetration, softening point, ductility, Brookfield viscosity, and segregation tests. Subsequently, a self-made asphalt fume detection device was used to analyze the emission patterns of H_2_S and VOCs in DCRMA. Finally, the deodorization mechanism of α-Amyl cinnamic aldehyde in the DCRMA system was revealed using two microscopic analysis methods: FM and FTIR.

## 2. Materials and Methods

### 2.1. Raw Materials

#### 2.1.1. Asphalt

To prepare CRMA and DCRMA for this experiment, 70# petroleum (Produced in Guangxi, China) asphalt was chosen as the matrix asphalt. The Chinese transportation industry standards (JTG 3410-2025 [17]), with the technical parameters listed in Table 1, and they are cited in the testing procedures. The test findings are in compliance with the norms.

#### 2.1.2. Crumb Rubber

For this study, CR with a 60-mesh Produced in Guangxi, China) particle size was selected, and Table 2 provides important technical details.

#### 2.1.3. Deodorant

In this study, α-Amyl cinnamic aldehyde (Produced in Guangxi, China) was chosen as the deodorizing agent for CRMA. Its fundamental performances are summarized in Table 3.

### 2.2. Preparation of DCRMA

Given that CR can adsorb the light components of asphalt at elevated temperatures, facilitating the full swelling of the CR and subsequently enhancing the performance of the asphalt, CR was initially added to the hot asphalt to undergo a pre-swelling process prior to the addition of the deodorant. Based on findings from previous studies [13], the CR content was set at 20% of the asphalt mass in this study. Based on this, α-Amyl cinnamic aldehyde was added to the CR-asphalt premix system at proportions of 0.5%, 1.0%, 1.5%, and 2.0% of the asphalt mass, respectively, to produce DCRMA. The preparation process for the deodorized rubberized asphalt in this study was carried out as follows:

(1) The CR was dried in an oven at 85 °C for 2 h.
(2) The base asphalt was melted by heating it to 135 °C in an oven. A specified mass of the base asphalt was then weighed and further heated to 180 °C. Once the temperature stabilized after adding CR, it was sheared at 2000 rpm for 30 min.
(3) Afterward, the deodorant was added at varying dosages, manually stirred with a glass rod for 5 min, and then sheared in a shear mixer at 5000 r/min for 1 h. The mixture was subsequently placed in an oven at 180 °C for 1 h to guarantee the complete swelling of CR within the asphalt. The final product, DCRMA, was obtained.

For comparison, CRMA without deodorant (containing 20% CR by asphalt mass) was prepared as the control group. The same preparation procedure was followed to guarantee consistency and dependability in the experimental outcomes.

### 2.3. Conventional Performance Testing of Asphalt

The asphalt was tested for penetration, ductility at 5 °C, softening point, Brookfield viscosity at 180 °C, and storage stability in compliance with the specification requirements in the Chinese standards JTG 3410-2025 [17] and CJJ/T 273-2019 [18].

### 2.4. Analysis of Deodorization Effect of Asphalt Fumes

#### 2.4.1. Detection Method for VOC Concentration in Asphalt Fumes

**Figure 1:**
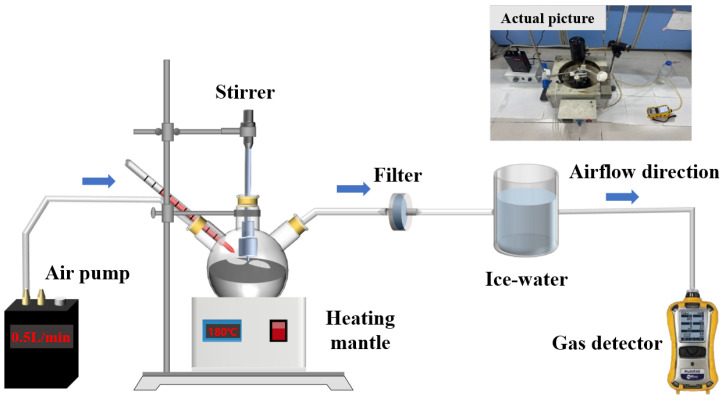
A self-made multi-index flue gas synchronous quantitative detection device.

Figure 1 is a diagram of the self-made device for producing and identifying asphalt fumes. The asphalt fume generation system includes a heating jacket, a stirrer, and a sealed three-neck flask. To ensure the circulation of fumes throughout the device, a gas pump with a flow rate of 500 mL/min is installed at the inlet of the fume generation system. A 200 g asphalt sample is placed in the three-neck flask, and the test temperature is set to 180 °C, consistent with the asphalt preparation temperature. The stirrer operates at a speed of 500 rpm. Prior to each fume test, the asphalt is pre-stirred for 1 min to facilitate the release of fumes, and the fume detection process is conducted over a duration of 30 min.

The asphalt fume detection component is composed of a real-time multi-gas monitor, the MultiRAE Lite Pumped. It is important to note that VOCs are measured by a photoionization detector (PID), which cannot differentiate between specific compounds. As a result, the detector provides only the total VOC concentration at any given time. The working range of the MultiRAE gas monitor for measuring VOCs is 0~1000 ppm.

Additionally, a glass fiber filter membrane and an ice-water bath are installed between the asphalt fume generation component and the detection component. The filter membrane is placed in a dedicated filter holder, while the ice-water bath is used to cool the gas to prevent damage to the detection instrument from high-temperature gases.

#### 2.4.2. Detection Method for H_2_S Concentration in Asphalt Fumes

**Figure 2:**
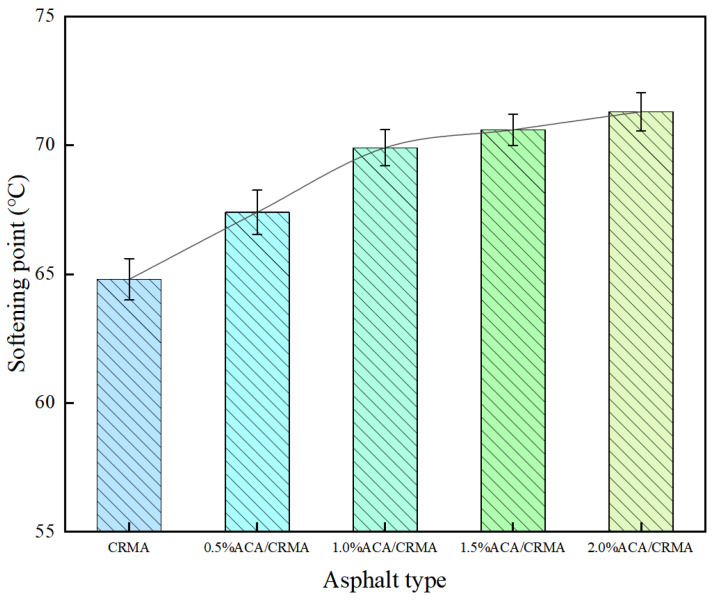
Softening-point test results of modified asphalt.

The apparatus depicted in Figure 2 is also used to determine the amount of H_2_S gas present in asphalt fumes. It should be noted that H_2_S is detected using an electrochemical sensor. The working range of the MultiRAE gas monitor for measuring H_2_S is 0~100 ppm.

#### 2.4.3. Analysis of Solid Particle Content in Asphalt Fumes

Prior to the fume detection process, the initial mass of the filter membrane (m_0_) is recorded. Following the detection, the filter membrane is removed, and its final mass (m_1_) is measured. The difference between m_1_ and m_0_ represents the mass change in the filter membrane, denoted as m (m = m_1_ − m_0_). The solid particle content in the flue gas is typically denoted by m [19].

### 2.5. FM Test

FM is a commonly used technique for assessing the compatibility of modifiers with asphalt [20]. In this work, FM was used to evaluate the dispersion and compatibility of the modifier within the asphalt matrix. Prior to the test, the asphalt was heated to achieve a fluid state. A tiny amount of the modified asphalt sample was sandwiched between a microscope slide and a cover slip, then heated in an oven at 100 °C and pressed repeatedly to ensure uniform distribution. When the asphalt was evenly dispersed between the glass slides, the sample preparation was considered complete.

### 2.6. FTIR Test

FTIR is commonly utilized to analyze the chemical composition of materials [21]. In this study, FTIR was used to investigate the changes in functional groups present in various asphalt samples. Prior to testing, the asphalt sample was dissolved in tetrahydrofuran to ensure total dissolution. The solution was then carefully dripped onto a transparent potassium bromide (KBr) wafer. After the tetrahydrofuran had fully evaporated, the prepared sample was ready for analysis.

## 3. Results and Discussion

### 3.1. Analysis of Conventional Performance Test Results of DCRMA

#### 3.1.1. Softening Point

The softening-point test results for several kinds of treated asphalt are shown in Figure 2. CRMA has a softening point of 64.8 °C. The softening points of CRMA with 0.5% to 2.0% ACA content are 67.4 °C, 69.9 °C, 70.6 °C, and 71.3 °C, respectively. When the ACA content is not more than 1.0%, the change in the softening-point value of the asphalt is more significant. Nevertheless, when the ACA content exceeds 1.0%, the trend of the softening-point value change becomes relatively flat. The maximum softening point occurs at an ACA content of 2.0%. The rise in the softening point suggests that the addition of ACA enhances the high-temperature stability of CRMA.

#### 3.1.2. Ductility

**Figure 3:**
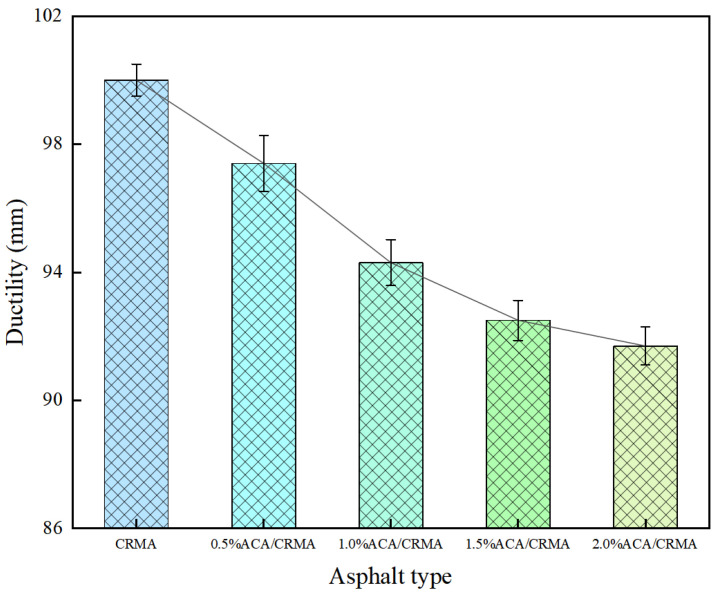
Ductility test results of modified asphalt.

Since CR does not completely dissolve in asphalt, using a higher stretching rate can produce a severe stress concentration effect, making CRMA prone to brittle fracture. The ductility test was performed on asphalt specimens in a 5 °C water bath at a stretching rate of 1 cm/min. From Figure 3, after the addition of the ACA modifier, compared with CRMA, the ductility of the asphalt showed a downward trend, decreasing by 2.6%, 5.7%, 7.5%, and 8.3%, respectively. Research suggests that a low dosage of ACA has a relatively limited impact on the low-temperature crack resistance of CRMA. However, with an increase in ACA, the ductility of the asphalt declines more significantly. This phenomenon is mainly attributed to two factors: First, the increase in ACA content leads to its self-aggregation within the asphalt system, forming polar microdomains that may disrupt the microscopic compatibility of the CR-asphalt system, thereby affecting the interfacial bonding between rubber and asphalt. Second, as the ACA dosage increases, the internal friction between macromolecules is intensified, significantly inhibiting the migration ability of the molecules, thereby enhancing the brittleness of the asphalt. Under constant tensile stress, this enhanced brittleness effect makes the asphalt more susceptible to fracture and destruction.

#### 3.1.3. Penetration

**Figure 4:**
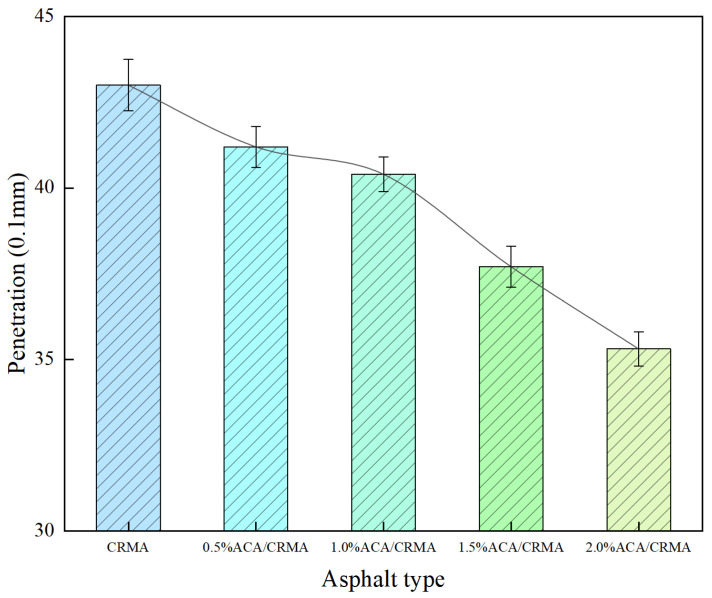
Penetration test results of modified asphalt.

Penetration tests were conducted on different asphalts at 25 °C, with the results shown in Figure 4. A lower penetration value corresponds to higher asphalt viscosity, which signifies greater resistance to deformation and improved suitability for high temperatures. The penetration of CRMA was 43.0 mm. After ACA was added at intervals of 0.5%, the penetration of DCRMA was 41.2 mm, 40.4 mm, 37.7 mm, and 36.3 mm, respectively. The addition of ACA was observed to increase the viscosity of the asphalt. The reason is that ACA reacts with asphalt molecules during the high-temperature modification process to form large molecules, increasing the ratio of large molecules and thereby raising the viscosity.

#### 3.1.4. Brookfield Viscosity at 180 °C

**Figure 5:**
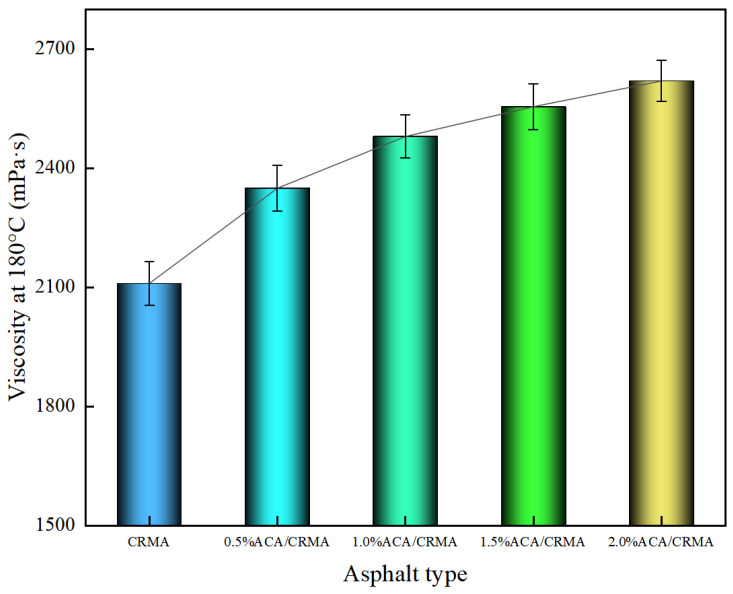
Viscosity test results of modified asphalt.

**Figure 6:**
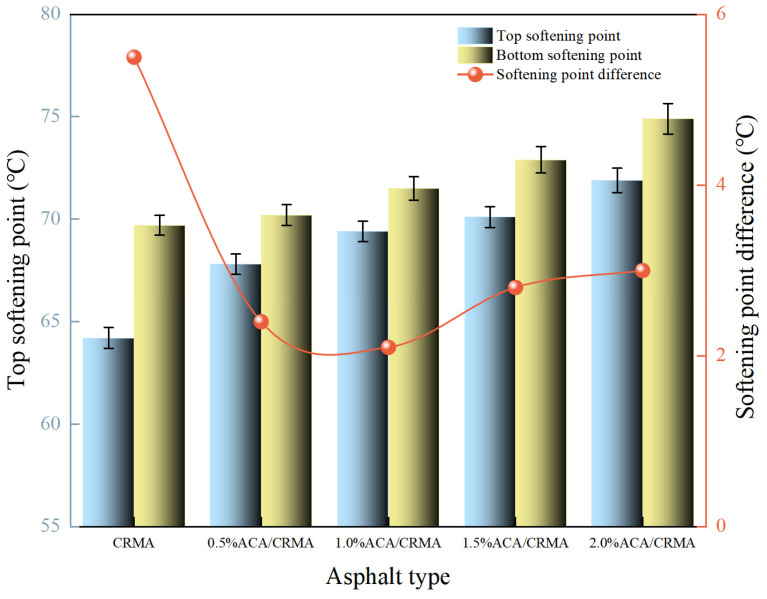
Segregation test results of modified asphalt.

Figure 5 shows the results of Brookfield viscosity experiments conducted on asphalt at 180 °C. During the preparation of CRMA, CR absorbs the lighter fractions of the asphalt and undergoes swelling, which increases the proportion of elastic components within the asphalt matrix. This process ultimately leads to an increase in the viscosity of the asphalt system [22]. As shown in Figure 6, ACA improves the viscosity of CRMA. Compared with CRMA, the viscosity of DCRMA increased by 11.4%, 17.5%, 21.1%, and 24.2%, respectively. ACA increases the viscosity of asphalt by reducing the ratio of light components, which increases the relative content of heavy components. This shift strengthens intermolecular forces, decreases asphalt fluidity, and ultimately raises its viscosity. Therefore, the asphalt shows improved resistance to deformation under shear stress.

The mixing temperature of the CRMA mixture is usually around 180 °C. The lower the viscosity, the easier it is for asphalt to coat the aggregate, and the more readily the asphalt can penetrate into the microcracks of the aggregate, resulting in better mixing effects. As shown in Figure 6, the asphalt with a 2.0% ACA content has the highest viscosity, indicating that it has superior high-temperature resistance to shear deformation. This aligns with the previous test conclusions obtained from penetration and softening-point tests. The 2.0% ACA/CRMA contains a higher proportion of heavy components, which gives it a higher viscosity.

#### 3.1.5. Storage Stability

The compatibility between CR and asphalt determines the storage stability of CRMA [23,24]. In this work, segregation tests were conducted to assess the storage stability of CRMA and DCRMA. The tests involved measuring the softening points of asphalt samples at the top and bottom of the test tubes. Figure 6 displays the outcomes of these examinations.

CRMA exhibits poor storage stability, with a softening-point difference of 5.5 °C. Under the influence of temperature and gravity, CR gradually settles to the bottom of the asphalt, causing a significant segregation phenomenon. After adding ACA, the softening-point difference decreased compared to CRMA, indicating an obvious improvement in the storage stability of the modified asphalt. The reason is that ACA can enhance the cross-linking of CR in asphalt, allowing CR to swell fully and distribute uniformly in the asphalt. However, when the ACA content exceeds 1.0%, the softening-point difference in the asphalt increases. This may be attributed to the increase in ACA content, which elevates the viscosity of the system and inhibits the migration and redispersion capability of rubber particles within the asphalt, ultimately leading to agglomeration due to localized high concentration. In addition, we consider that ACA molecules, as a polar material, introduce excessive polar groups into the asphalt system when the ACA content surpasses an optimal level. This alters the overall polarity balance of the asphalt system and induces the aggregation of polar components, thereby triggering microphase separation, which manifests as the reagglomeration of rubber.

### 3.2. Analysis of Deodorization Effect of Asphalt Fumes

#### 3.2.1. Analysis of VOC Concentration Detection Results

**Figure 7:**
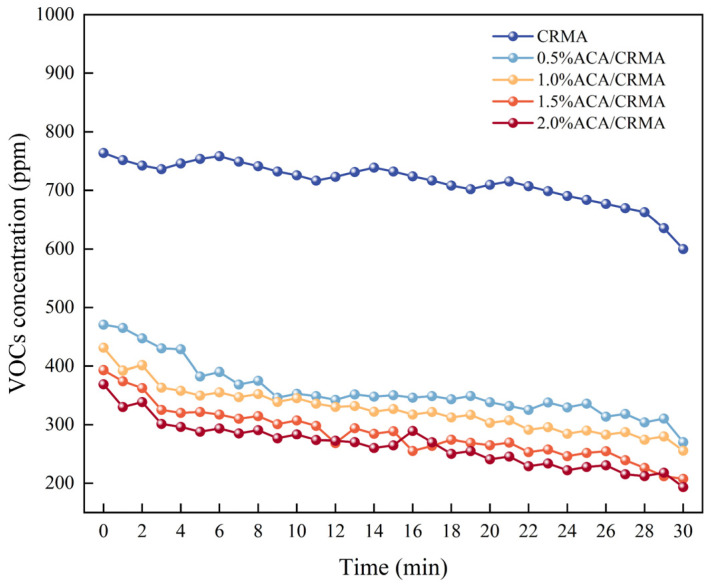
Variation in VOC concentration in asphalt flue gas.

**Figure 8:**
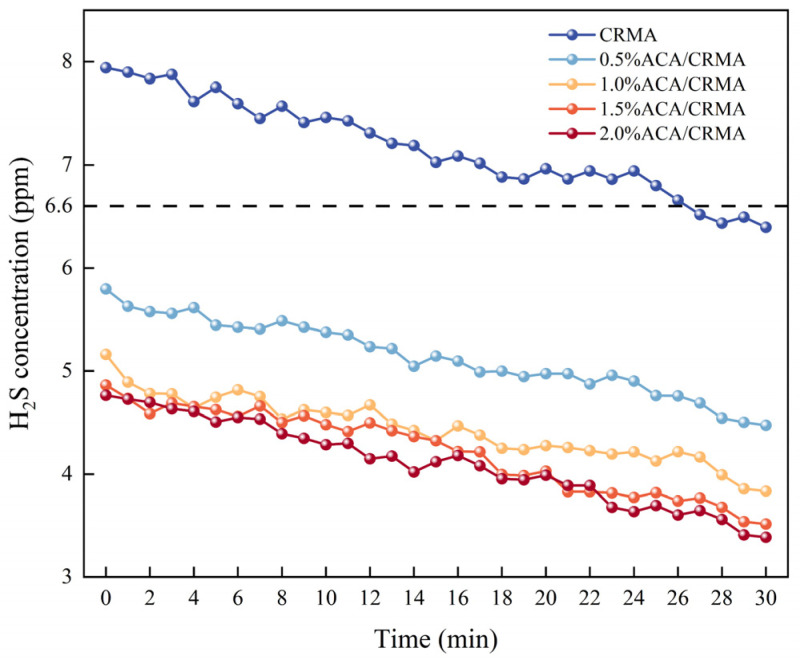
Variation in H2S concentration in asphalt flue gas.

The VOC concentration detection results of various asphalts are presented in Figure 7. As shown in Figure 8, the VOC concentration in asphalt fumes fluctuates within the detection time range. The maximum VOC concentration of CRMA is 761 ppm, with an average VOC concentration of 714 ppm within 30 min. In contrast, the average VOC concentrations of the modified asphalts with added ACA are 358 ppm, 325 ppm, 284 ppm, and 265 ppm, respectively, representing a reduction of 49.9%, 54.5%, 60.2%, and 62.9% compared to CRMA. This can be attributed to the addition of ACA, which prominently improves the high-temperature stability of asphalt. This lowers the content of VOCs by reducing the diffusion of light components and efficiently suppressing the thermal breakdown of asphalt molecules. This result indicates that ACA can reduce the emission of odorous and irritating gases from CRMA to some extent, thereby mitigating the environmental and health hazards posed by the fumes generated during CRMA heating.

#### 3.2.2. Analysis of H_2_S Concentration Detection Results

The detection results of H_2_S concentration in different asphalts are shown in Figure 8. Crumb rubber undergoes vulcanization reactions at high temperatures, producing large amounts of H_2_S gas, which is the main odor-causing composition in CRMA fumes [25]. As shown in Figure 8, the overall trend of H_2_S gas concentration in different asphalts is a decrease with increasing heating time. According to the National Health Commission of China (GBZ 2.1-2019), there can be no more than 6.60 parts per million of H_2_S in the air at a workplace [6]. Using the instantaneous maximum concentration as the evaluation standard for H_2_S gas concentration [12], the instantaneous maximum concentration of H_2_S in CRMA fumes is 7.9 ppm. The instantaneous maximum concentrations of H_2_S in the modified asphalts with added ACA are 5.8 ppm, 5.2 ppm, 4.9 ppm, and 4.8 ppm, respectively. Compared with CRMA, the addition of ACA reduces the instantaneous maximum concentration of H_2_S in CRMA by 26.6%, 34.2%, 38.0%, and 39.2%. The instantaneous maximum concentrations of H_2_S in DCRMA with different ACA contents all meet the regulatory requirements, suggesting that ACA has an apparent inhibitory effect on H_2_S gas in CRMA and effectively removes the main odor-causing component from CRMA.

#### 3.2.3. Analysis of Solid Particle Content Results

**Figure 9:**
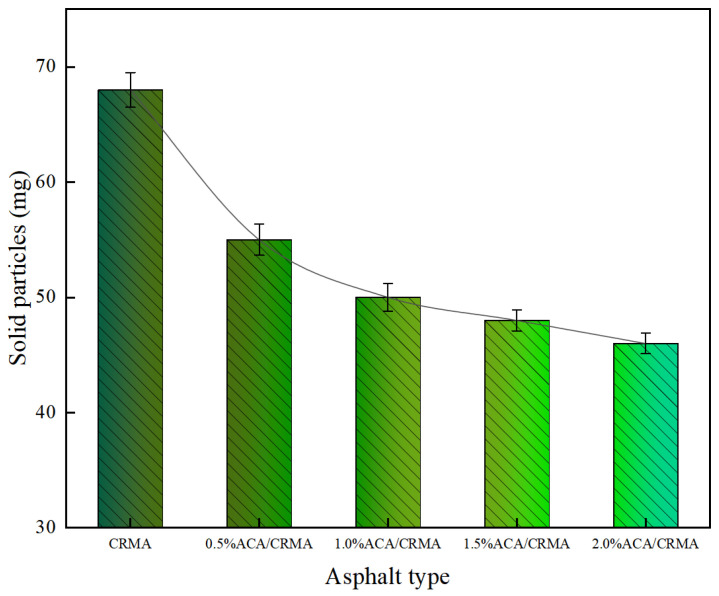
Change in weight (m) of filter membrane for asphalt fume collection.

Figure 9 illustrates the weight change (m) of the filter membrane after fume gathering for five different types of asphalt. As shown in the figure, the weight of the filter membrane increased for all asphalt samples after 30 min of fume collection, with each type of asphalt exhibiting varying degrees of weight change. A larger weight change indicates a higher content of solid particles in the asphalt fumes. The weight change m for CRMA reached 68.2 mg. Compared with CRMA, the weight change m for DCRMA with added ACA decreased by 19.4%, 26.7%, 29.6%, and 32.6%, respectively. It is evident that the higher the ACA content, the smaller the weight change m, indicating that the addition of ACA effectively reduces the solid particle content produced by asphalt heating. It is important to note that when the ACA concentration exceeds 0.5%, the decrease in weight change m tends to level off, suggesting that further increasing the ACA content has a limited effect on reducing m.

### 3.3. Analysis of Deodorization Mechanism

#### 3.3.1. Phase Analysis of DCRMA Based on FM

**Figure 10:**
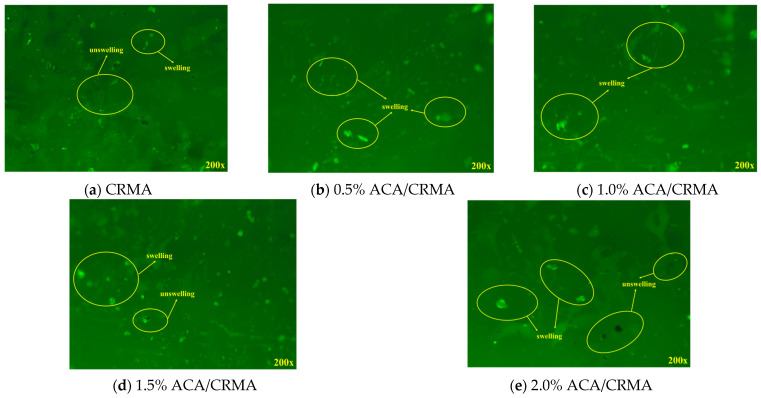
Fluorescence micrograph of asphalt.

FM scanning was conducted at a uniform magnification of 200×, and the resulting FM images of the different asphalt samples are presented in Figure 10. The CRMA fluorescence picture displays a lot of black, unswollen CR particles, with some agglomeration. After adding ACA, more fluorescent spots appear in the images, indicating fewer unswollen CR particles. This suggests that ACA can enhance the swelling level of CR in asphalt, promoting the production of a more robust structure between CR and asphalt. As a result, DCRMA exhibits better high-temperature stability than CRMA, which can restrict the decomposition of light components in CRMA during high-temperature heating, reducing the release of foul-smelling gases and thereby achieving the deodorization goal.

Notably, the bidirectional effect of ACA on the dispersion state of CR observed under fluorescence microscopy is closely related to its molecular structure. The ACA (α-amylcinnamaldehyde) molecule consists of a polar aldehyde group, an aromatic ring, and a non-polar amyl side chain, conferring amphiphilic properties. At low dosages (≤1.0%), ACA tends to migrate to the interface between CR particles and asphalt. Its polar aldehyde group may interact with polar components in asphalt, such as asphaltenes and resins, or with active sites on the CR surface; meanwhile, the non-polar amyl side chain remains compatible with the asphalt matrix. This interfacial bridging effect reduces the interfacial tension between CR and asphalt, promoting the swelling and uniform dispersion of CR (as shown in Figure 10c). When the ACA dosage exceeds 1.0%, adsorption on the CR particle surface approaches saturation. The excess ACA molecules, unable to be accommodated at the interface, undergo self-aggregation within the asphalt matrix driven by dipole interactions and potential hydrogen bonding between their polar aldehyde groups, forming polar microdomains. These microdomains exhibit significant polarity differences from the bulk asphalt, disrupting the polarity balance of the system and inducing reagglomeration of CR particles around these microdomains (as shown in Figure 10d,e), ultimately leading to microphase separation. This phenomenon aligns with the polarity matching theory in multiphase polymer systems.

#### 3.3.2. Chemical Functional Group Analysis of DCRMA Based on FTIR

**Figure 11:**
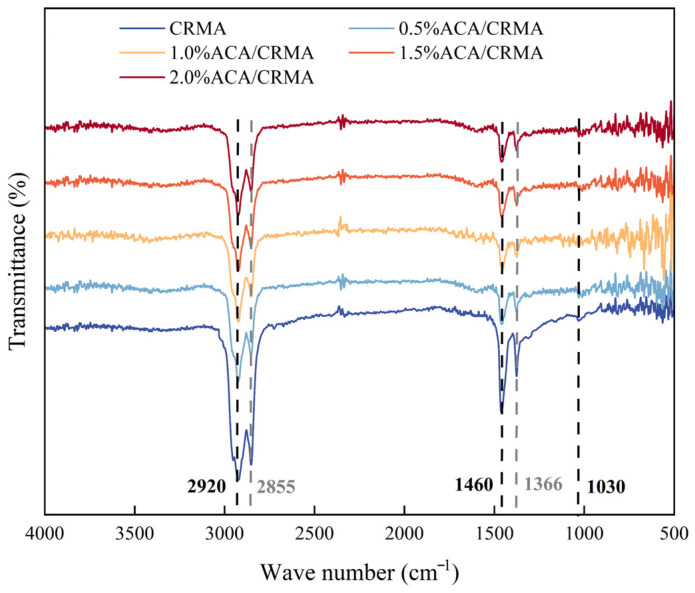
FTIR test results.

The infrared spectroscopy test results of different asphalts are shown in Figure 11. In this study, FTIR analysis was performed to distinguish the functional groups of five different types of asphalt, aiming to investigate whether the addition of ACA induces chemical reactions with CRMA. As shown in Figure 11, the FTIR spectra of the five asphalts are essentially the same. The peak at 1030 cm^−1^ is due to the vibration of the S=O bond, with CRMA showing the highest peak, indicating that CRMA is more prone to aging [26]. Additionally, the peaks at 2855 cm^−1^ and 2920 cm^−1^ are linked to the symmetric and asymmetric stretching vibrations of C-H bonds in aliphatic hydrocarbons, respectively, whereas the peaks at 1366 cm^−1^ and 1460 cm^−1^ show the bending vibrations of C-H bonds [27]. The FTIR test results indicate that, in comparison to CRMA, the four DCRMA samples did not display any new peaks. This suggests that the interaction between ACA and CRMA is predominantly a physical mixing process rather than a chemical reaction. Comparing the characteristic peak intensities of different asphalts reveals that CRMA has the highest peak intensity, suggesting that the addition of ACA can reduce the content of certain substances in CRMA, with the corresponding decrease in peak intensity. This finding is related to the inhibitory effect of ACA on the foul-smelling gases in CRMA.

## 4. Conclusions and Future Work

This study systematically evaluated the effects of ACA as a deodorant on the conventional performance, fume emissions, and microstructure of CRMA, leading to the following key conclusions:

(1) ACA exerts a biphasic regulatory effect on the performance of CRMA. An appropriate dosage of ACA significantly enhances the softening point and viscosity of CRMA, improving its high-temperature deformation resistance, while slightly reducing its low-temperature ductility. When the ACA content exceeds 1.0%, self-aggregation of ACA occurs within the asphalt, disrupting the compatibility of the CR-asphalt system and leading to rubber reagglomeration and reduced storage stability. Therefore, 1.0% is identified as the optimal dosage for balancing performance and stability.
(2) ACA significantly inhibits the emission of hazardous gases from CRMA. With increasing ACA content, the average concentration of VOCs in asphalt fumes (reduced by 49.9–62.9%), the instantaneous maximum concentration of H_2_S (reduced by 26.6–39.2%), and the content of solid particulate matter (reduced by 19.4–32.6%) all exhibited continuous decreases. By promoting rubber swelling and stabilizing the system, ACA effectively mitigates occupational health risks during construction.
(3) The mechanism of ACA is primarily governed by physical modulation. Fluorescence microscopy revealed that an appropriate ACA dosage facilitates the swelling and uniform dispersion of rubber in asphalt, whereas excessive ACA induces rubber reagglomeration due to interfacial saturation and self-aggregation effects. Fourier transform infrared spectroscopy detected no significant formation of new chemical bonds, indicating that ACA primarily functions through physical means such as interfacial modification and system stabilization, rather than altering the chemical structure of the asphalt.
(4) Future research can further analyze the specific elements of VOCs in asphalt emissions and explore the synergistic effects of ACA with other deodorants to promote its large-scale application in practical engineering.

## Figures and Tables

**Table 1:** Technical index of 70 # petroleum asphalt.

Technical Index		Unit	Test Results	Standard
Penetration (25 °C)		0.1 mm	65.0	60~80
Ductility (10 °C)		cm	32.4	—
Softening point (T_R&B_)		°C	47.5	≥46
Viscosity (135 °C)		mPa·s	366	<3000
RTFOT	Mass loss	%	−0.56	≤±0.8
	Residual penetration ratio (25 °C)	%	82.0	≥61

**Table 2:** 60 mesh crumb rubber technical indicators.

Test Items	Moisture Content/%	Metal Content/%	Fiber Content/%	Ash Content/%	Rubber Hydrocarbon Content/%
Technical index	<1	<0.03	<1	≤8	≥42
Test results	0	0.02	0.38	6	48

**Table 3:** Basic properties of α-Amyl cinnamic aldehyde.

Items	Physical Form	Boiling Point (°C)	Solubility	Chemical Structure
Results	Yellow oily liquid	288	Soluble in acetone	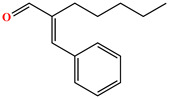

## Data Availability

The original contributions presented in this study are included in the article. Further inquiries can be directed to the corresponding author.
